# Nanobodies and their derivatives: pioneering the future of cancer immunotherapy

**DOI:** 10.1186/s12964-025-02270-4

**Published:** 2025-06-05

**Authors:** Haixia Li, Quan Zhou, Nan Cao, Chenghao Hu, Jincheng Wang, Yu He, Shan Jiang, Qi Li, Miao Chen, Li Gong, Ming Luo, Xinzhou Deng, Zhiguo Luo

**Affiliations:** 1 Department of Clinical Oncology, Hubei Provincial Clinical Research Center for precision Diagnosis and Treatment of liver cancer, Taihe Hospital, Hubei University of Medicine, Shiyan, Hubei 442000 P.R. China; 2 Department of Traditional Chinese Medicine, Renmin Hospital, Hubei University of Medicine, Shiyan, Hubei 442000 P.R. China; 3 Key Laboratory of Cancer Therapy Resistance and Clinical Translational Study, Shiyan, Hubei 442000 P.R. China; 4 Department of Respiratory, Taihe Hospital, Hubei University of Medicine, Shiyan, Hubei 442000 P.R. China; 5 Department of Biochemistry, School of Basic Medical Sciences, Hubei University of Medicine, Shiyan, Hubei 442000 P.R. China

**Keywords:** Nanobody, Cancer immunotherapy, CAR-T therapy, Immune checkpoint inhibitors, Immune cell engaging antibodies

## Abstract

Cancer immunotherapy, which boosts the immune system to recognize and attack malignant cells, has revolutionized traditional cancer treatment paradigms. Approaches such as chimeric antigen receptor T cell (CAR-T) therapy and immune checkpoint inhibitors (ICIs) have demonstrated promising therapeutic outcomes, leading to the approval of numerous immuno-oncology agents by the US Food and Drug Administration (FDA) over the past few decades. Immuno-oncology agents, mainly based on conventional full-length antibodies or their derivatives, are widely used in cancer immunotherapy. However, their large size, unwanted immunogenicity, poor solubility, complex molecular architectures, and limited tumor penetration pose significant challenges that must be addressed. Nanobodies, which are single-domain antibody fragments originating from the variable regions of camelid heavy-chain immunoglobulins, represent the smallest known antigen-binding fragments. In addition to their small size (~ 15 kDa), nanobodies possess a range of advantageous properties, including high stability, strong specificity and affinity for target antigens, low immunogenicity, and cost-effective production. Nonetheless, their short serum half-life and lack of Fc-mediated functions may limit efficacy, which can be addressed by Fc fusion, albumin binding, or multivalent construct design. These properties enable nanobodies to support multifunctional constructs, such as bispecific CARs, nanobody-secreting CARs, dual ICI-containing molecules, and trispecific immune cell-engaging antibodies. In recent years, a growing number of nanobody-based immuno-oncology agents have progressed into preclinical and clinical trials, with several products approved by the US FDA and China’s National Medical Products Administration for cancer therapy. In this review, we explore the unique properties of nanobodies and provide a comprehensive summary of recent preclinical and clinical advancements in nanobody-based immuno-oncology agents, with a focus on their applications in CAR-T cells, ICIs, and immune cell-engaging antibodies. Through their unique capacity to integrate innovative molecular engineering with translational clinical development, nanobody-based therapeutics are poised to revolutionize current paradigms in cancer immunotherapy.

**Graphical Abstract:**

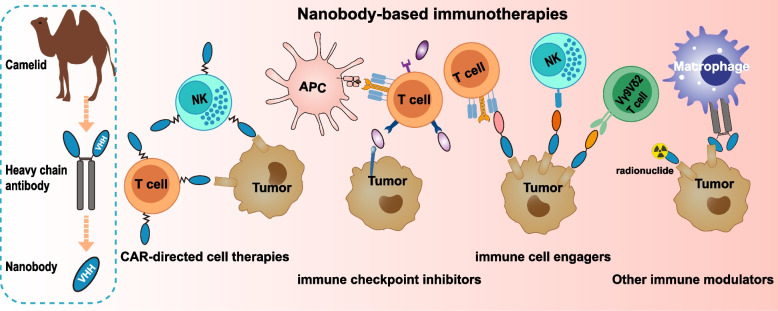

## Introduction

Cancer is a leading cause of death globally, with the burden of cancer-related morbidity and mortality continuing to rise at an alarming rate. The GLOBOCAN 2022 estimates indicate that around 20 million new cancer diagnoses and nearly 10 million cancer-related deaths occurred globally. By 2050, the global number of new cancer cases is projected to increase by 77% compared to 2022, reaching an estimated 35 million cases [1]. Effective cancer prevention, early diagnosis, and innovative treatment strategies are therefore critical to mitigating this growing public health challenge.

Currently, the primary modalities for cancer treatment include surgery, radiotherapy, chemotherapy, targeted therapy, and immunotherapy [2]. While surgery, radiotherapy, chemotherapy, and targeted therapy directly target tumor cells for removal or destruction, immunotherapy represents a fundamentally distinct approach. By harnessing the power of the immune system to recognize and eliminate cancer cells, immunotherapy aims to induce durable anti-tumor responses and maintain long-term remission [3, 4]. The concept of cancer immunotherapy traces its origins to 1891 when William B. Coley pioneered using streptococcal organisms to treat a patient with inoperable sarcoma [5, 6]. This groundbreaking work laid the foundation for decades of research into immune-based strategies for cancer treatment. Owing to the promising clinical outcomes demonstrated by immune checkpoint inhibitors (ICIs) and chimeric antigen receptor T cell (CAR-T) therapy, cancer immunotherapy was heralded as the “breakthrough of the Year” in 2013 by Science magazine [7]. James P. Allison and Tasuku Honjo were awarded the 2018 Nobel Prize in Physiology or Medicine for their seminal discoveries in immune checkpoint regulation, which paved the way for novel therapeutic strategies to combat cancer by inhibiting negative immune regulation. In recent decades, immunotherapeutic approaches have become fundamental to both oncological research paradigms and clinical cancer management strategies. To date, a variety of immuno-oncology agents have been approved by the US Food and Drug Administration (FDA), offering new hope to cancer patients. As the field advances, innovative immunotherapeutic approaches are poised to further expand the frontiers of cancer treatment.

## Cancer-immunity cycle and synthetic immunology

A comprehensive understanding of the interplay between cancer and the immune system is essential for the development of more effective immuno-oncology agents. The immune system is a highly sophisticated and tightly regulated network that plays a pivotal role in defending the body against disease and maintaining homeostasis. The process by which the immune system mounts an effective antitumor response, known as the Cancer-Immunity Cycle, involves a series of coordinated steps. It begins with the release of neoantigens from malignant cells, which are captured and processed by antigen-presenting cells (APCs), primarily dendritic cells (DCs). Upon maturation, DCs migrate to draining lymph nodes, presenting the antigens to naïve T cells via major histocompatibility complex (MHC)-I and MHC-II molecules, leading to their differentiation into cytotoxic T cells (CTLs). CTLs then infiltrate the tumor microenvironment, recognize tumor cells in an MHC-dependent manner, and induce tumor cell death. The release of antigens from dying cells initiates subsequent cycles, amplifying the antitumor immune response [8–10]. Despite the critical role of T cells as the primary effector cells in antitumor immunity, tumors have evolved multiple mechanisms to evade immune detection and destruction. These include the downregulation or loss of MHC-I presentation and the suppression of DC function, which hinder effective antigen presentation and T-cell activation [11]. Notably, the density and functional status of tumor-infiltrating lymphocytes (TILs) have been shown to correlate strongly with the prognosis of cancer patients, underscoring the importance of enhancing TIL activity in cancer immunotherapy [12, 13].

Synthetic immunology, an emerging interdisciplinary field that integrates principles of immunology and synthetic biology, offers innovative approaches to address these challenges. By employing engineered biological systems, synthetic immunology enables the rational modulation and reprogramming of immune responses. In cancer therapy, synthetic immune strategies hold significant potential by facilitating the artificial activation and targeted redirection of T cells toward tumors, thereby enhancing therapeutic efficacy [14–16]. Antibodies, as central molecules of the immune system, play a critical role in recognizing, targeting, and eliminating malignant cells. Over the past four decades, antibody-based strategies have evolved significantly, with a growing emphasis on enhancing the ability of immune cells to attack tumor cells and inhibit their progression. This paradigm shift has catalyzed a surge in the development and clinical application of antibody-based immune-oncology agents, marking a new era in cancer treatment.

## Nanobodies: promising antibody alternatives with unique physicochemical characteristics

Conventional monoclonal antibodies (mAbs) have been widely utilized in cancer therapy [17]. However, their limitations, including large molecular size, structural complexity, and high production costs, have driven the search for alternative antibody formats and derivatives. Among these, single-chain variable fragments (scFvs) and nanobodies (Nbs) have emerged as the most extensively studied antibody formats [17, 18].

ScFvs, derived from monoclonal antibodies, retain antigen-binding specificity and affinity while offering reduced molecular size (~25 kDa), enabling clinical applications such as CAR-T cells and bispecific T cell engagers (BiTEs) [17, 19, 20]. However, their rodent origin, low human sequence homology, and potential immunogenicity limit therapeutic efficacy [20, 21]. Structural instability, linker-dependent functionality, and aggregation-prone hydrophobic residues further constrain their utility [19, 22–24]. These limitations underscore the need for innovative antibody formats to address these challenges.

**Fig. 1:**
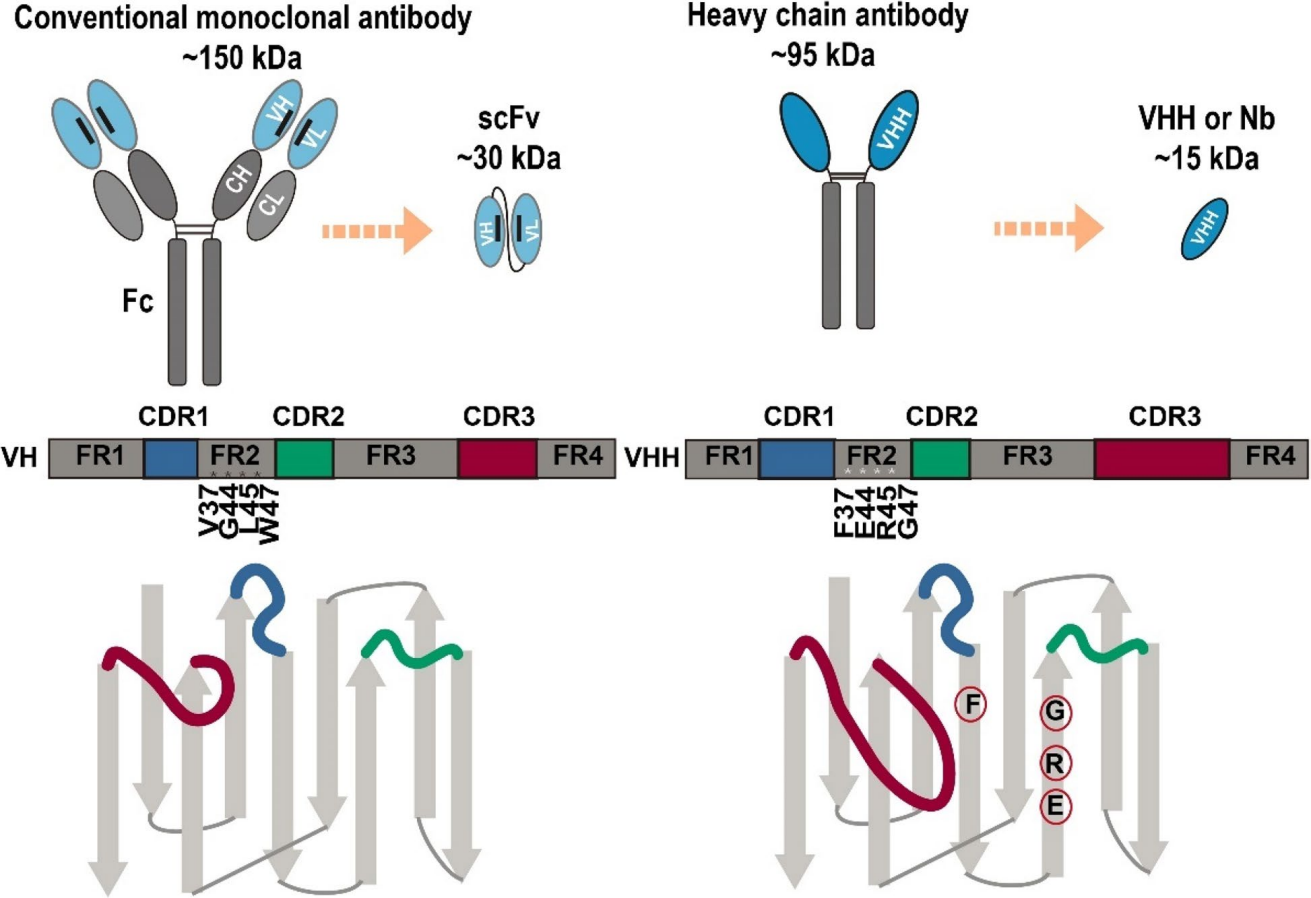
Schematic comparison of the structure of scFv and nanobodies. Conventional scFvs, derived from monoclonal antibodies, consist of a VH and a VL connected by a flexible linker. In contrast, nanobodies are composed of the variable domain of heavy chain of heavy-chain antibody. Compared with conventional VH domains, nanobodies feature a longer CDR3, which allows for high-affinity antigen binding. Additionally, four conserved hydrophobic residues (Val37, Gly44, Leu45 and Trp47) in FR2 are replaced by more hydrophilic ones (Phe37, Glu44, Arg45 and Gly47) in nanobodies

ScFvs have been regarded as the smallest antibody fragments capable of antigen binding for decades until the discovery of nanobodies revolutionized this perspective. In 1993, Hamers-Casterman et al. accidentally identified a unique heavy-chain antibody (HcAb) in camel serum, which lacked light chains and the first constant domains of the heavy chains [25]. The variable antigen-binding fragment at the N-terminal of HcAbs is referred to as a single-domain antibody (sdAb), also known as the variable domain of heavy chain of heavy-chain antibody (VHH) or nanobody (Fig. 1). With a diameter of approximately 2.5 nm, a length of about 4 nm, and a molecular weight of ~15 kDa, nanobodies are half the size of scFvs and represent the smallest known antigen-binding antibody fragments to date [26, 27]. Structurally, nanobodies resemble the variable heavy chain (VH) domains of human or murine antibodies, containing four conserved framework regions (FRs) and three hypervariable complementarity-determining regions (CDRs). Unlike conventional antibodies, which bind antigens through six CDRs, nanobodies rely on only three CDRs for antigen recognition. The CDRs of nanobodies are non-canonical and extended, enabling broad paratope diversity in the absence of variable light chains (VLs). Notably, nanobody has a particularly long CDR3 which allows for high-affinity antigen binding. And the V (variable) D (diversity)-J (joining) recombination brings significant sequence variation to CDR3, further enhancing its diversity. In nanobodies, CDR3 contributes to the construction of the antigen-binding site and paratope diversity, making it crucial for antigen recognition and specificity. CDR1 sequences are highly similar within certain nanobody families but vary in phylogenetically distant families, likely contributing to divergent epitope recognition [28–33]. Another key distinction between the VH of conventional antibodies and nanobodies is the substitution of four highly conserved hydrophobic residues in FR2. These residues, which mediate interactions with the VL in conventional antibodies, are typically replaced by smaller, polar, and more hydrophilic residues (Phe37, Glu44, Arg45, and Gly47 in Kabat numbering) in nanobodies. This substitution enhances the solubility and stability of nanobodies while reducing their tendency to aggregate [29, 34]. Conventional antibodies typically form paratopes with cavities, grooves, or flat surfaces to bind small molecules, linear peptides, or large protein epitopes, respectively. In contrast, nanobodies exhibit a convex binding surface, enabling them to interact with cryptic epitopes or cavities that are often inaccessible to conventional antibodies [30, 35, 36]. Nanobodies could be generated as intact molecules, retaining high affinity and specificity. Their equilibrium dissociation constants can reach nanomolar or even picomolar levels, making them suitable for a wide range of applications [37]. Furthermore, nanobodies share a high degree (75–90%) of sequence identity with human VH3 domains, contributing to their low immunogenicity in monomeric form. This low immunogenicity supports the potential for repeated dosing of monomeric nanobody-based drugs [20, 38, 39]. Additionally, nanobodies can be expressed in prokaryotes (such as *Escherichia coli*), enabling cost-effective large-scale production [40]. Despite these advantages, nanobodies exhibit rapid renal clearance and lack Fc regions, leading to short serum half-life and absent Fc-mediated effector functions, potentially limiting therapeutic efficacy. These challenges are mitigated by molecular engineering, including Fc fusion, albumin-binding domain incorporation, and multivalent or multispecific constructs, to extend circulation, restore effector activity, and enhance efficacy [41–43].

**Fig. 2:**
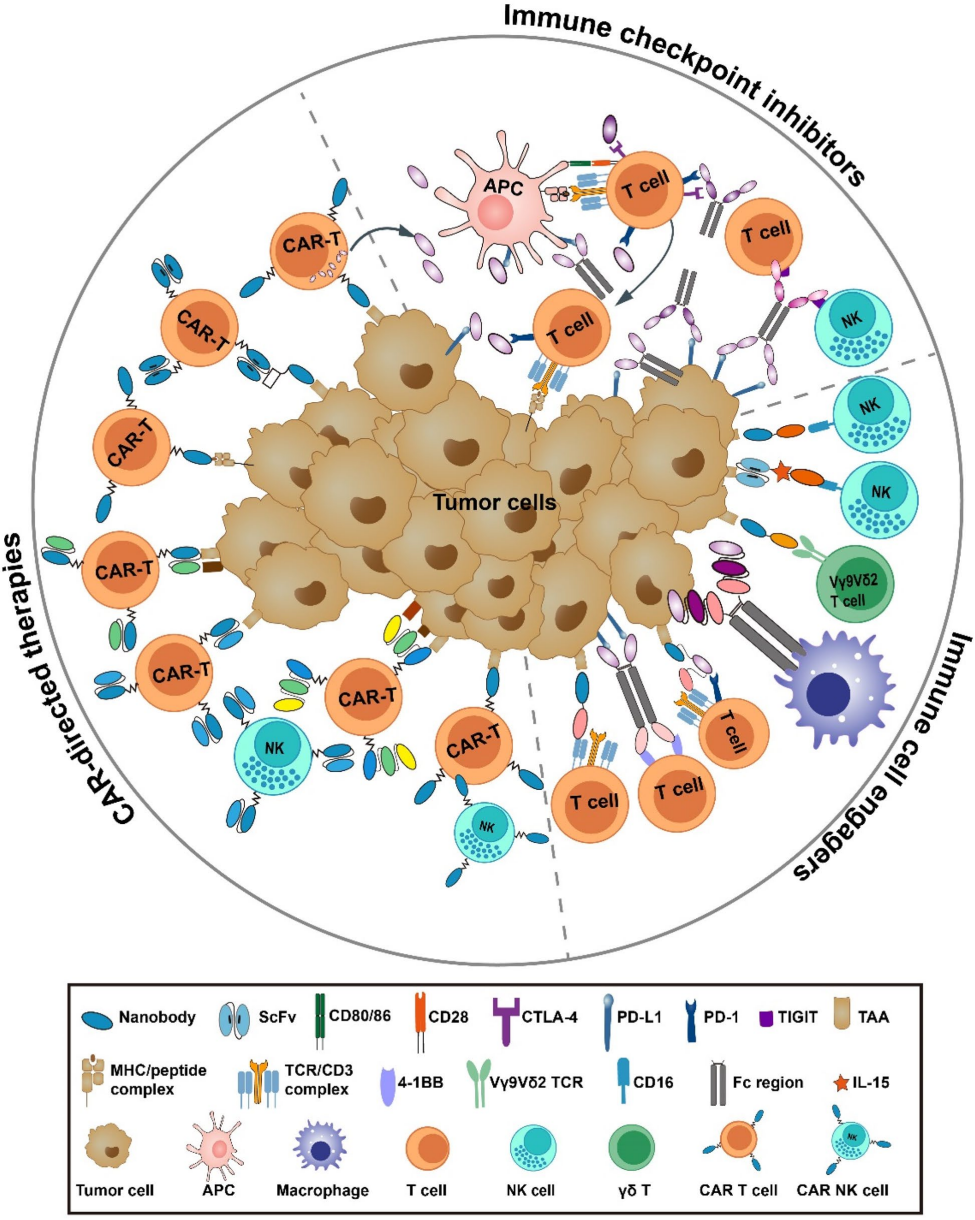
Overview of nanobody-based strategies for enhancing immune recognition and targeting tumor cells. CAR-modified T cells could directly recognize and attack tumor cells in an MHC-independent manner. ICIs enhance T cell-mediated antitumor responses by blocking inhibitory signals, thereby “releasing the brakes” on T cell activity. Immune cell-engaging antibodies facilitate targeted activation and cytotoxicity of T cells, NK cells and Vγ9Vδ2 T cells within the TME

The characteristics of small size, structural stability, good solubility, low aggregation tendency, high affinity, and minimal immunogenicity have made nanobodies an invaluable supplemental tool for developing next-generation antibody-based immune-oncology agents [17, 18]. In recent years, a variety of nanobodies targeting diverse antigens have been generated, and nanobody-based agents have demonstrated promising therapeutic efficacy in both preclinical and clinical cancer treatments. This comprehensive review examines the evolving landscape of nanobody-based cancer immunotherapies, focusing on their translational development and clinical implementation in chimeric antigen receptor platforms, immune checkpoint modulation, and immune cell-redirecting therapeutic strategies (Fig. 2).

## Nanobody-based CAR-directed immunotherapies

CAR technology, first pioneered by Eshhar et al. in 1989, revolutionized T cell-based immunotherapy by enabling MHC-independent recognition of tumor-associated antigens (TAAs) [44]. Early CAR designs fused antibody-derived VH and VL chains to T cell receptor (TCR) domains, creating chimeric receptors capable of targeting specific antigens like 2,4,6-trinitrophenyl (TNP). Subsequent advancements led to the development of first-generation CARs, incorporating an extracellular scFv derived from an antibody, a hinge region, a transmembrane region, and an intracellular signal transduction domain (the CD3ζ subunit of the TCR/CD3 complex) [45–47]. While these CAR-T cells demonstrated antigen-specific cytotoxicity, their limited persistence and efficacy in early clinical trials highlighted the need for improved designs [48]. The integration of costimulatory domains (such as CD28 or 4-1BB) into second-generation CARs markedly improved T cell proliferation, persistence, and antitumor activity relative to their first-generation counterparts [49–51]. Subsequent modification of CARs, including third- and fourth-generation constructs, further improved anticancer efficacy by adding multiple costimulatory domains or transgene expression cassettes like IL-12 [52, 53].

**Fig. 3:**
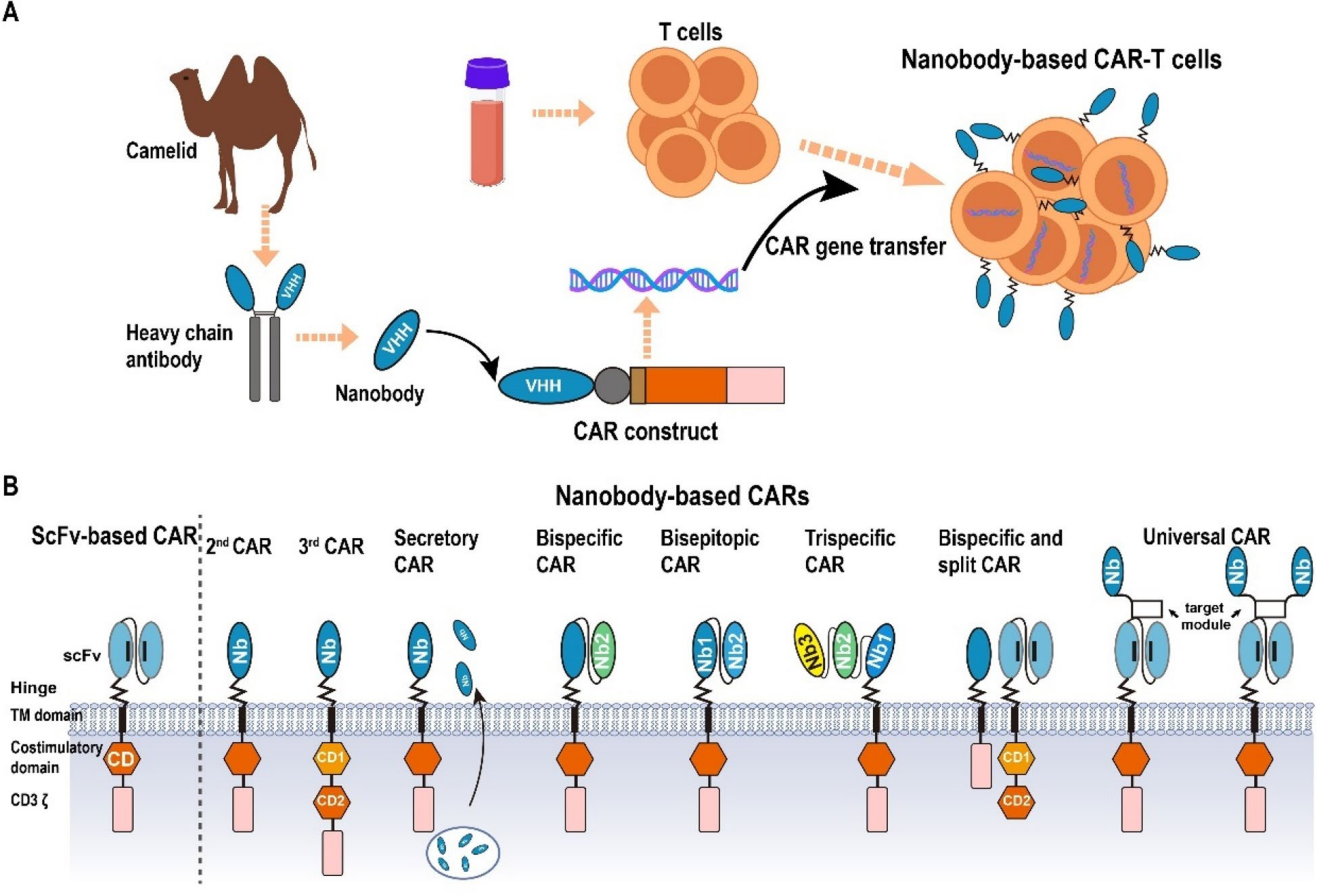
Schematic overview of the production process and structural formats of nanobody-based CAR constructs. **A** Nanobody sequences derived from camelids are genetically incorporated into CAR constructs and then introduced into T cells, generating nanobody-based CAR-T cells. **B** Structure of CAR molecules, which typically consist of an extracellular scFv, a hinge domain, a transmembrane region and cytoplasmic domains including a CD3ζ chain and a costimulatory signaling domain. As an alternative to scFvs, nanobodies have been utilized to design second-generation CARs, third-generation CARs, Bi/Tri-specific CARs, biepitopic CARs and universal CARs

Among these, second-generation CAR-T therapies targeting CD19 or B cell maturation protein (BCMA) have shown impressive results in treating hematologic malignancies [54–58]. To date, the FDA has approved six CAR-T therapies, four targeting CD19 and two targeting BCMA [59, 60]. Despite these clinical successes, scFv-based CAR-T cells face main challenges: (1) limited stability and aggregation propensity, even in humanized scFvs [22, 24]; (2) immunogenicity due to murine-derived sequences, triggering anti-CAR immune responses and rapid clearance [61–65]; and (3) risk of CAR clustering and tonic signaling, leading to T cell exhaustion [66–68]. Unlike scFv, nanobodies exhibit low immunogenicity and lack domain swapping, making them a promising alternative [69]. Nanobody-based CAR-T cells are typically generated by replacing the conventional scFv with an antigen-specific nanobody, most commonly screened from immune camelid libraries, while synthetic and naïve camelid libraries are also utilized in preclinical research [70]. The nanobody is fused with CAR structural domains to construct a CAR vector, which is subsequently used to transduce T cells via viral delivery, followed by in vitro expansion and infusion [69, 71] (Fig. 3A). The utilization of nanobodies as antigen-targeting domains in CAR-T cells offers a promising approach to overcome the aforementioned limitations, prompting extensive research efforts in developing nanobody-based CAR-T cells in recent years (Fig. 3B).

### Single-target CAR-T cells

Bakhtiari et al., for the first time, reported a functional second-generation CAR construct engineered with an anti-MUC1 nanobody. MUC1 CAR-modified Jurkat cells were selectively activated by MUC1-positive MCF7 cells, along with significant cytolytic activity against these target cells. The study indicated that the nanobody-based CAR-T cells (Nb CAR-T) could be specifically activated by target tumor antigens, showing comparable activation patterns to scFv-based CARs. The findings established the technical feasibility of nanobody-based CAR constructs and highlighted their potential as promising alternatives for CAR-T cell development [72]. Inspired by this study, Moghimi et al*.* isolated nanobodies specific for tumor-associated glycoprotein (TAG)−72 and human epidermal growth factor receptor (HER2), subsequently generating both second-generation (CD28-CD3ζ) and third-generation (CD28-OX40-CD3ζ) CAR constructs. T cells (CCRF-CEM or Jurkat T cells) armed with oligoclonal Nb-CARs showed enhanced antigen-binding specificity and superior proliferation capacity, cytokine secretion profiles, and cytotoxic potential, compared to monoclonal counterparts [73, 74]. Although these nanobody-based CARs have demonstrated functional efficacy in vitro, their clinical translation necessitates further investigation in human primary T cells and systematic in vivo evaluation. With the development of genetic engineering technology and phage display technologies, it is possible to obtain nanobodies with high affinity and specificity against a multitude of antigens. Technological progress has facilitated the development of numerous nanobody-based CAR-T cells targeting a diverse array of tumor-associated antigens. These include targets expressed in hematological malignancies (such as BCMA, CD19, CD20, CD13, CD38, CD33, CD72, and CD7), solid tumors (such as PSMA, FGFR4, and GPC2) and tumor microenvironment (such as VEGFR2, EIIIB, PD-L1, and CD105) [69, 71, 75–83]. Preclinical studies have consistently validated the antigen-specific tumor recognition and eradication potential of these nanobody-engineered CAR-T cell platforms across both in vitro and in vivo experimental models.

Target antigen selection is pivotal to CAR-T cell therapy success. TAAs in hematological malignancies, particularly CD19 and BCMA, have been the most extensively investigated targets in CAR-T cell therapy research. Nasiri et al. generated second-generation CD19 CAR-T cells by incorporating a nanobody-based binding moiety. In comparison with their scFv-based counterparts, the CD19 VHH-CAR-T cells exhibited similar expansion rate, cytotoxic behavior, and cytokine (IFN-γ, IL-2, and TNF-α) secretion ability [84]. Then, they humanized an anti-CD19 nanobody in silico approaches and constructed CARs using both humanized and native nanobodies to assess the impact of humanization. The two CAR-T cells showed comparable properties and antitumor activity [85]. An anti-BCMA nanobody with high binding affinity and specificity was developed from an immunized alpaca and constructed a CAR incorporating the humanized anti-BCMA nanobody, 4-1BB, and CD3ζ signaling domains. These BCMA CAR-T cells demonstrated potent cytotoxicity against MM.1S cells (high BCMA expression level) both in vitro and in vivo murine experiments. In a cohort of 34 multiple myeloma patients undergoing BCMA-directed CAR-T cell therapy, clinical outcomes at a median follow-up of 12.5 months demonstrated an 88.2% overall response rate, including a 55.9% rate of stringent complete remissions. The one-year overall survival rate was 78.8%, and no specific toxicities were observed. Long-term follow-up data from the ongoing trials are needed to further compare its clinical efficacy with the approved BCMA-targeted scFv-based CAR-T therapies [86]. Besides, a separate study showed that BCMA CAR-T cells were well-tolerated and efficacious for managing multiple myeloma patients with hepatitis B virus infection when combined with antiviral drugs [87].

Unlike hematologic malignancies, CAR-T therapy for solid tumors faces significant challenges, including antigen heterogeneity and immunosuppressive tumor microenvironment (TME) [88]. Given the shared characteristics of the tumor microenvironment in solid tumors, targeting the microenvironment may serve as an effective therapeutic strategy to overcome these challenges. Immune checkpoint molecules and stromal cells within the tumor microenvironment represent potential targets for CAR-T cell therapy. Nanobody-mediated CAR-T cell platforms directed against either PD-L1 or the fibronectin splice variant EIIIB exhibited significant antitumor efficacy and improved tumor immune infiltration in immunocompetent murine models of solid malignancies. B7-H3 (CD276) is an immune checkpoint molecule that is overexpressed in various tumors, as well as blood vessels and stromal cells in TME [89]. Its high expression is associated with tumor immune evasion, enhanced metastatic potential, and poor clinical prognosis, highlighting its potential as an ideal therapeutic target [90]. Meeus et al. developed B7-H3 NbCAR-T cells for glioblastoma treatment, which demonstrated potent activation and specific cytotoxic activity even at low antigen levels [91]. Another study identified three nanobodies, C4 and B12 binding to the IgC domain, and G8 binding to the IgV domain of B7-H3 from a large nanobody library. These nanobody-based B7-H3 CAR-T cells exhibited cytotoxicity against multiple solid tumors in vitro, including neuroblastoma (NB), pancreatic ductal adenocarcinoma (PDAC), triple-negative breast cancer and lung adenocarcinoma in an antigen-dependent manner. Notably, CAR-T cells targeting the IgC domain showed superior and persistent antitumor activity in NB and PDAC mouse models [92]. These B7-H3 NbCAR-T cells also demonstrated superior antitumor effects in vitro compared to traditional antibody-based CAR-T cells in the treatment of pediatric glioblastoma. In vivo studies are underway to evaluate the efficacy of B7-H3 CAR-T cells in orthotopic glioblastoma models, using both immunocompromised and syngeneic mouse models [93, 94]. Epstein-Barr Virus-Specific T Cells armed with nanobody-based B7-H3-targeting CARs could not only inhibit the growth of B7-H3^+^ solid tumor cells, such as colorectal cancer, non-small cell lung cancer, triple-negative breast cancer, and gastric cancer cells, but also mitigate the immunosuppressive tumor microenvironment by targeting B7-H3^+^ myeloid-derived suppressor cells. These findings underscore the versatility of B7-H3 NbCAR-T cell therapy in addressing both tumor cells and immune evasion mechanisms [95]. CD105 is overexpressed on neoangiogenic endothelial cells and cancer cells. CD105 NbCAR-T cells significantly inhibited the growth of hepatocellular carcinoma across established cell line-based and clinically relevant patient-derived tumor models [80].

It is well known that each component of the CAR structure, as well as their combination, influences its functionality, including antibody affinity, hinge length, transmembrane domain, and co-stimulatory domain [68, 96]. Nanobody-based CAR optimization has also been investigated. MSLN is a promising tumor-associated antigen overexpressed in various solid cancers, however, CAR-T cells engineered with high-affinity MSLN-specific receptors have demonstrated target-specific cytotoxicity against both malignant and normal tissues expressing the antigen in preclinical investigations and clinical trials [97, 98]. To address this, Yang et al. identified an MSLN-specific nanobody named JZQ-B4 VHH that could cross-react with human and mouse MSLN, and then generated 4 variants with reduced affinity using alanine scanning via a yeast surface display system. These variants and the parental JZQ-B4 VHH were converted into CARs to measure their risk of off-tumor toxicity in human tumor xenograft models. Affinity-reduced CARs, compared to their high-affinity counterparts, mediated enhanced tumor-specific T cell redirection, leading to improved safety profiles of CAR-T cell therapy. This study established a promising strategy for preclinical safety evaluation of CAR-T cell therapy [99]. Furthermore, McComb et al. systematically screened and characterized seven distinct nanobodies targeting multiple epitopes of CD22, which were subsequently engineered into second-generation CAR constructs for functional evaluation with varying binding affinities. Among these constructs, only CD22 CAR-T cells incorporating a membrane-proximal epitope-targeting nanobody coupled with a CD8 transmembrane domain exhibited favorable therapeutic efficacy with reduced off-target toxicity than the conventional scFv-based CAR-T cells [100]. It is suggested that the CAR binding epitope, rather than the antibody affinity, plays a crucial role in determining nanobody-based CAR function. Li N et al. isolated a nanobody targeting the membrane-distal epitope of GPC1 overexpressed in pancreatic cancer. Then, GPC1 CARs with various hinge domains and transmembrane domains were constructed to explore the optimal design. Their investigation revealed that the integration of an IgG4 hinge (the shorter variant) with a CD28 transmembrane domain facilitated CAR dimerization, substantially augmenting the antitumor performance of GPC1-targeted CAR-T cells against pancreatic malignancies, particularly under conditions of low GPC1 antigen expression, as demonstrated in cellular assays and murine xenograft studies [101]. However, another study revealed that the nanobody-based third-generation VEGFR2 CAR-T cells with a longer hinge space were more efficient in activation, cytokine release, and tumor cell lysis [102]. Nanobody-engineered CAR-T cells directed against membrane-proximal EGFR demonstrated that the hinge domain truncation, while compromising overall cellular functionality, significantly improved tumor-specific selectivity [103]. These studies demonstrated that epitope location determines the necessity of hinge domains, and established hinge modification as a programmable approach for CAR optimization.

Conventional strategies for tumor treatment involve target identification followed by antibody screening to engineer CAR-T cells. However, some researchers have explored alternative strategies to discover novel targets and antitumor agents by first screening tumor-specific nanobodies, followed by identification of the interacting cell surface antigens. Feng et al. first identified VHH1, a nanobody specifically binding to neuroendocrine tumors (NETs), from a large naïve nanobody library. Then, they demonstrated CDH17 to be the surface antigen bound by VHH1. Next experiments proved CDH17 overexpressed in NET and gastrointestinal cancers, suggesting CDH17 a potential target for NET and gastrointestinal cancer treatment. The VHH1-based second- and third-generation CAR-T cells were developed, and the third-generation CAR-T cells exhibited enhanced cytotoxicity towards NETs and gastrointestinal cancers in a CDH17-dependent manner in various tumor models without causing off-tumor toxicity [104].

### Dual/multi-target CAR-T cells

Tumor antigen escape remains a major challenge for cancer immunotherapy, including CAR-T therapy [105]. Bi- or tri-specific CAR-T cells, which target two or more antigens simultaneously, may reduce tumor relapse and treatment failure caused by low antigen expression. Nanobodies offer distinct advantages over scFv in advanced CAR design due to their single-domain structure, eliminating VH/VL mis-paring risks associated with multi-specific scFv-based CARs [69, 106]. Moreover, their compact size minimizes constraints on viral vector packaging capacity, facilitating efficient CAR delivery [107, 108]. De Munter et al. first engineered a bispecific CAR construct featuring a tandem fusion of CD20-specific and HER2-specific nanobodies to mediate dual antigen recognition. The bispecific CAR-T cells could be activated by either CD20 or HER2. However, in vitro assays did not show their superior cytotoxicity over monospecific CAR-T cells. Further optimization of nanobody-based bispecific CAR structures and in vivo evaluation of their therapeutic efficacy are warranted [109]. High-affinity and specific nanobodies targeting CD30 and CD5 were screened and then engineered into bispecific CAR-T cells, termed NbCD30-CD5-CAR-T cells. These CAR-T cells demonstrated superior tumor suppression in T-cell lymphoma compared to NbCD30-CAR-T cells, NbCD5-CAR-T cells, and scFv-based bispecific CD30-CD5-CAR-T cells [110]. In another study, anti-CD19 and CD20 nanobodies with high specificity and affinity were selected from a naïve phage Nb display library to generate bispecific CAR-T cells. The CD19/CD20 bispecific CAR-T cells showed robust cytotoxic activity against Raji and Daudi cells, which endogenously express both CD19 and CD20. Additionally, they exhibited superior killing efficacy against primary tumor cells from acute lymphoblastic leukemia (ALL) patients compared to CD19 or CD20 NbCAR-T cells [111]. Significantly, the CD19/CD20 bispecific nanobody-based CAR-T therapeutic approach has progressed to phase 1 clinical evaluation for patients with R/R B-cell lymphoma (NCT03881761), though results are not available yet. Addressing the therapeutic constraints associated with single-antigen CD19-directed CAR-T approaches in refractory B-cell neoplasms, Zhou et al. presented a novel trispecific NbCAR-T (LCAR-AIO) targeting CD19, CD20, and CD22. In vitro and in vivo results demonstrated that LCAR-AIO exhibited superior cytotoxicity, cytokine production, and tumor-killing efficacy, even against CD19-negative tumors. Additionally, LCAR-AIO showed enhanced T-cell expansion and persistence in vivo [112]. Another nanobody-based tri-specific Tandem CAR (TanCAR) T-cell targeting CD33, CD123, and CLL1 were designed to address antigen-negative escape and intra-tumor heterogeneity in acute myeloid leukemia (AML). The TanCAR-T cells exhibited strong cytotoxicity, proliferation, and cytokine production against AML cells expressing single antigens in vitro [113]. The clinical application of CD33- and CD123-targeting CAR-T cells has been limited due to their on-target off-tumor toxicity against hematopoietic stem cells (HSCs) and normal tissues. To mitigate these side effects, researchers have developed bispecific and split CAR-T cells, termed BissCAR-T cells. In this approach, CD13 Nb fused with CD3ζ targets CD13 on both HSCs and leukemic stem cells (LSCs), while the anti-TIM3 scFv fused with CD28 and 4-1BB specifically binds to TIM3, which is uniquely expressed on LSCs. This dual-targeting mechanism ensures that BissCAR-T cells are exclusively activated by LSCs, reducing the risk of off-tumor toxicity [75]. These approaches provide potential solutions for treating malignancies with antigen heterogeneity and reducing toxicity.

In addition to bi- and tri-specific CAR-T cells, CAR-T cells targeting different epitopes of a single TAA simultaneously may also be an attractive strategy to mitigate the risk of antigen escape and enhance clinical efficacy. Ciltacabtagene autoleucel (commonly referred to as cilta-cel, LCAR-B38M, or JNJ-4528) is an engineered CAR-T therapy featuring a biepitopic targeting domain composed of two anti-BCMA nanobodies, thereby enhancing both target specificity and binding avidity for BCMA-expressing malignant cells [56]. In a phase 1 clinical study involving 57 patients with R/R multiple myeloma, LCAR-B38M CAR-T cells revealed a best overall response rate of 88%, a complete response rate of 68%, 14-month median response duration, and 15-month median duration of disease control, alongside a manageable safety profile. These results indicated that LCAR-B38M CAR-T cells might achieve comparable clinical efficacy at lower cell doses and reduced toxicity compared with bb-2121 (also called idecabtagene vicleucel) [114]. A phase 1b/2 open-label study conducted in the U.S. reported deep and durable responses in patients with R/R multiple myeloma following a single infusion of cilta-cel. Among 97 treated patients, 65 achieved stringent complete response, with an overall response rate of 91.2%−99.4%, a 1-year overall survival rate of 89%, and a progression-free rate was 77% [56]. Cilta-cel demonstrated excellent clinical efficacy in clinical trials and was approved by the FDA for treating R/R multiple myeloma in adults [115]. Cilta-cel represents a significant milestone as the first FDA-approved nanobody-incorporated CAR-T therapy targeting BCMA. Lu et al. engineered two different CD7-specific nanobodies into a second-generation CAR (dVHH NS7CAR). The dVHH NS7CAR-T cells demonstrated enhanced antigen-binding specificity, affinity, and anti-tumor efficacy in xenograft mouse models compared to single nanobody- and scFv-based CD7 CAR-T cells in xenograft models. Initial phase I trial data (NCT04938115) showed promising efficacy and safety in AML patients [116].

### TCR-like CAR-T cells

The therapeutic scope of CAR-T is constrained by the limited repertoire of TAAs expressed on the cell surface, as a significant proportion of these antigens are intracellular and thus inaccessible to conventional targeting approaches [117]. These intracellular antigens are proteolytically cleaved into short peptides, which are then presented on the cell surface as MHC/peptide complexes, recognizable by TCRs [118]. The development of TCR-mimic CAR-T cells broadens the therapeutic horizon of CAR-T technology, enabling the targeting of previously inaccessible intracellular tumor antigens. Li et al. developed TCR-like NbCAR T cells with two TCR-like nanobodies screened from the immune nanobody phage display library. These TCR-like Nb CAR T cells could kill tumor cells specifically by recognizing the HLA-A2/GPC3_144-152_ complex or HLA-A2/WT1_126-134_ complex on tumor cells [117]. Human papillomavirus (HPV) infection contributes to HPV-related cancers, and the viral antigens E6 and E7 serve as potential targets for cancer immunotherapy. Nanobodies (F5 and G9) specific for the monomer of E6_29-38_ peptide complexed with HLA-A2 were identified and cloned into second-generation CARs. The F5 CAR-T cells not only specifically killed HLA-A2/E6_29-38_ complex-positive cervical cancer cells by activating NFAT and NF-κB signaling pathways, but also inhibited tumor growth in mouse models [119].

### Universal CAR-T cells

Despite the remarkable efficacy of CAR-T therapy, on-target off-tumor toxicity remains a primary concern [120]. Conventional CAR-T cells, once transferred into patients, can’t be controlled or regulated, potentially leading to lethal side effects. To regulate CAR-T cells in patients, a modular CAR platform technology, termed UniCAR, was developed. Unlike conventional CARs, UniCARs recognize a common peptide epitope on target modules (TMs) rather than directly binding to TAAs. TMs consist of a small peptide epitope and an antigen-binding moiety, serving as a molecular bridge to redirect UniCAR-T cells against TAAs. This design allows UniCAR-armed T cells to be switched on only upon TM administration, enabling precise targeting of different TAAs by simply changing TMs [121]. Albert et al. constructed nanobody-based monovalent and bivalent anti-EGFR TMs, which efficiently redirected UniCAR-T cells to recognize and lyse EGFR-positive tumor cells in vitro and in vivo. Importantly, TMs could dissociate from UniCAR-TM complexes and be rapidly cleared, providing a controllable safety mechanism. This rapid clearance may create a therapeutic window, allowing UniCAR-T cells to selectively target tumor cells with high antigen expression while sparing healthy tissues with low antigen expression [122, 123]. Building on the switchable CAR concept, He et al. generated a nanobody-based switch by fusing a CD13-specific nanobody (Nb157) with a peptide neo-epitope (PNE) to regulate CAR-T cell activity. In xenograft mouse models, the switchable CAR-T cells demonstrated comparable antitumor efficacy to conventional Nb157 CAR-T cells but exhibited reduced toxicity toward normal hematopoietic stem cells at low switch doses. Similar results were observed in PDX models, highlighting the potential of this approach [124]. By utilizing different nanobody-based switches, the activity of modular CAR-T cell platforms can be precisely engineered to achieve patient-specific therapeutic responses with temporal control, offering a promising strategy to minimize side effects while maintaining therapeutic efficacy.

### CAR-NK cells

CAR-T therapy for targeting malignant T-cells faces challenges, such as a lack of normal CAR-T cell sources and fratricide of CAR-T cells, NK cells present a viable therapeutic alternative for T-cell malignancies. NK cells also exhibit distinct advantages over T cells, such as their capacity to kill tumor cells in an MHC-independent manner, lower risks of graft-versus-host-disease (GVHD) and cytokine release syndrome (CRS), and broader availability [125, 126]. CD5 and CD7 are potential targets for T-cell malignancy treatment, and their nanobodies have been generated [126, 127]. Zu et al. cloned the CD5 nanobody into a fourth-generation CAR construction together with a CD28 hinge, a transmembrane, and intracellular domain, a CD3ζ signaling domain, and the human IL-15 gene. These CD5 CAR-NK cells demonstrated enhanced antitumor activity against CD5^+^ malignant T cells in vitro and in xenograft mouse models compared to CD5 CAR-NK cells without IL-15 [126]. NK-92MI cells expressing either monovalent CD7 NbCAR or bivalent dCD7 NbCAR demonstrated consistent and potent cytotoxicity against both cell lines and primary tumor cells of T-ALL [127]. Beyond T-cell malignancies, CAR-NK therapy has also been developed to target surface antigens and intracellular antigens of other tumors. Verhaar et al. generated CAR-NK-92 cells based on MICA-specific nanobodies. The MICA-targeting CAR-NK-92 cells exhibited MICA-dependent cytotoxicity against MICA-positive cells and demonstrated efficient localization to lung metastases in mouse models [125]. In another study, TCR-like CAR-NK-92 cells exhibited potent cytotoxic activity against both melanoma cell lines and primary melanoma cells through specific targeting of the gp100/HLA-A2 complex.

## Nanobody-based immune checkpoint inhibitors

**Fig. 4:**
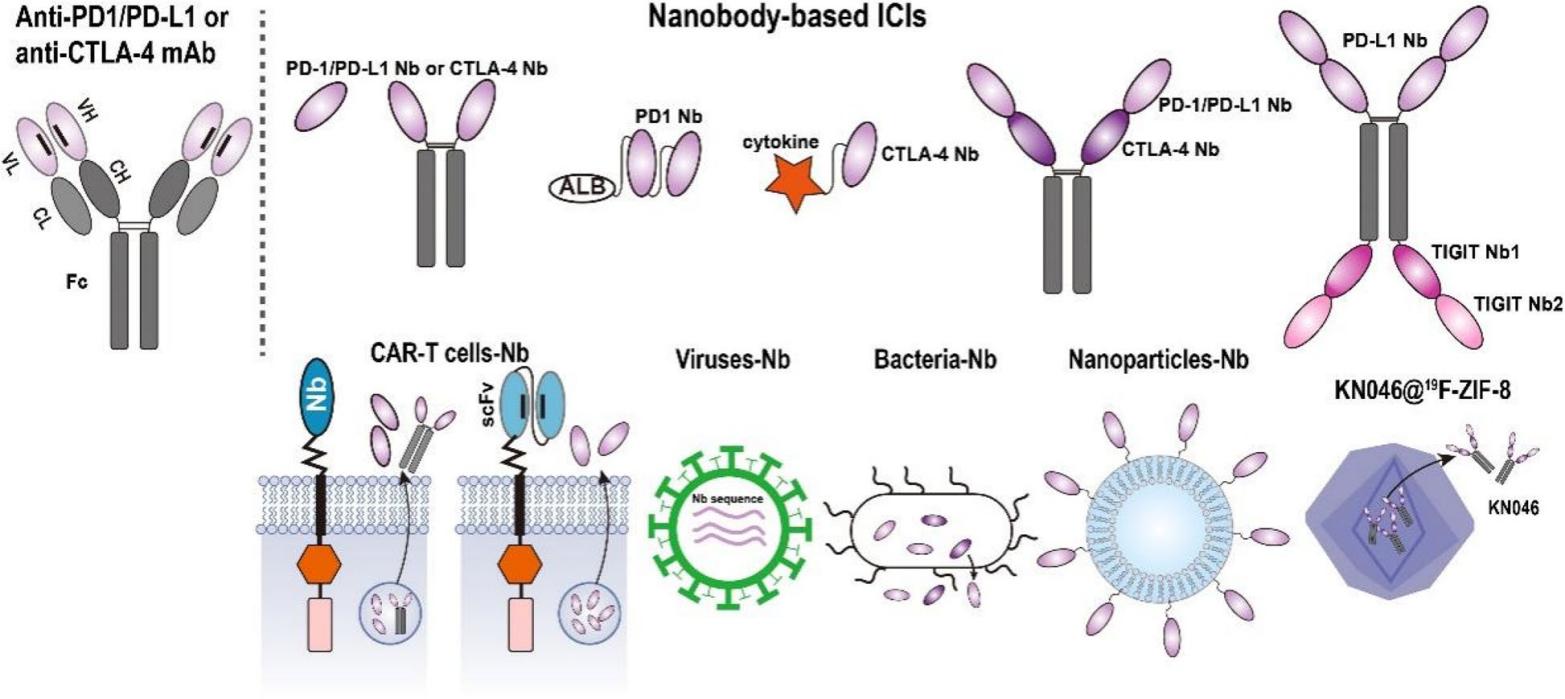
Schematic overview of diverse nanobody-based formats for immune checkpoint inhibitors. Conventional ICIs are typically monoclonal antibodies, however, nanobody-based proteins have also been developed to block immune checkpoint pathways. Various ICI delivery systems are illustrated

Immunosuppression is a hallmark of cancer progression, enable tumors to evade immune surveillance, especially by suppressing tumor antigen-specific T cells [128]. A key mechanism underlying this immune evasion involves the activating of immune checkpoint pathways within the TME. Immune checkpoints, such as cytotoxic T-lymphocyte-associated protein 4 (CTLA-4) and programmed cell death protein 1 (PD-1), are inhibitory receptors expressed on immune cells that regulate immune responses to maintain homeostasis and prevent autoimmunity under physiological conditions. However, tumors exploit these pathways by aberrantly upregulating immune checkpoint molecules (e.g., CTLA-4 and PD-1 on T cells) or their ligands (e.g., PD-L1 on tumor cells), thereby dampening antitumor immunity. Immune checkpoint inhibitors (ICIs) counteract this immunosuppression by blocking checkpoint interactions, effectively "releasing the brakes" on T cells to restore antitumor activity [129–132]. The approval of Ipilimumab, an anti-CTLA-4 monoclonal antibody, by the FDA in 2011 for advanced melanoma marked a pivotal milestone in cancer immunotherapy. Subsequent clinical development has established PD-1/PD-L1 axis inhibitors as clinically validated therapeutic agents across multiple solid tumor indications, revolutionizing treatment paradigms for malignancies such as melanoma, non-small cell lung cancer, and renal cell carcinoma [133–137]. Conventional ICIs, primarily monoclonal antibodies, face inherent limitations due to their large size, which restricts tumor penetration and necessitates intravenous infusion. Leveraging their small size, superior tissue penetration, and modular architecture, nanobodies enable the engineering of multifunctional constructs, addressing critical challenges associated with traditional immune checkpoint blockade strategies (Fig. 4).

### Novel ICIs

Through camel immunization strategies, Lu et al. successfully generated high-affinity nanobodies targeting CTLA-4 and PD-1, which were subsequently employed in a comprehensive series of experimental investigations. These nanobodies demonstrated the ability to enhance the antitumor activity of CD8^+^ T cells both in vitro and in vivo when used in conjunction with DC/tumor fusion cell vaccine [138, 139]. To further augment T-cell-mediated immune responses, the researchers generated fusion proteins by linking the CTLA-4 nanobody to interleukin-12 (IL-12) or the human IgG1 Fc domain. As a result, CTLA-4 Nb-IL-2 and CTLA-4 Nb-Fc elicited more robust and sustained immune reactions in CD8^+^ T cells across hepatocellular carcinoma, breast cancer, and melanoma mouse models [140, 141]. In a related approach, Yang et al. modified liposomes (LPS) with anti-CTLA-4 nanobodies to improve target-binding ability. When stimulated with a DC/tumor fusion vaccine and LPS-CTLA-4 Nb, CD8^+^ T cells exhibited enhanced immune responses and persistence in various mouse models [142]. Similarly, Broos et al. investigated the function of K2, an anti-PD-L1 nanobody, and found that it effectively blocked PD-L1 on DCs, thereby promoting the activation and proliferation of antigen-specific CD8^+^ T cells primed by DCs [143]. Further advancements include the development of an anti-PD-1 nanobody (Nb97). The recombinant protein Nb97-Nb97-human serum albumin exhibited significantly enhanced efficacy in blocking the PD-1/PD-L1 pathway compared to the Nb97-Fc [144]. Additional anti-PD-1 and PD-L1 nanobodies have also been reported in subsequent studies [145, 146]. Given the critical role of CKLF (chemokine-like factor)-MARVEL transmembrane domain containing protein 6 (CMTM6) in stabilizing PD-L1/PD-1 interaction through the formation of a CMTM6-PD-L1 complex, Jia et al. generated a CMTM6-specific nanobody using a high-quality immunized nanobody phage library. The nanobody significantly disturbed PD-L1/PD-1 signaling, alleviating T-cell suppression in vitro, and inhibited tumor growth in syngeneic tumor models [147]. Notably, a high-affinity anti-PD-L1 nanobody was identified, exhibiting potent antitumor activity comparable to durvalumab, an FDA-approved anti-PD-L1 mAb. Building on this discovery, Alphamab Oncology developed envafolimab (KN035) by fusing the anti-PD-L1 nanobody with an Fc fragment, designed to treat various solid tumors. Envafolimab offers advantages in terms of safety, convenience, and patient compliance due to its unique design [41]. In phase 1 and 2 clinical studies, subcutaneous administration of envafolimab monotherapy proved to be safe and effective in patients with advanced deficient MisMatch Repair or microsatellite instability-high (dMMR/MSI-H) solid tumors [148, 149]. Based on these promising results, the FDA granted orphan-drug designation to envafolimab for the treatment of advanced biliary tract cancer, and it has been approved in China for the treatment of dMMR/MSI-H advanced solid tumors. Envafolimab is the first approved subcutaneous PD-L1 antibody and the first nanobody-based ICI for cancer treatment globally [150].

### Bispecific ICIs

Although dual blockade of the PD-1 and CTLA-4 immune checkpoint pathways demonstrates enhanced therapeutic outcomes, this approach faces challenges including Fc-mediated adverse effects and immunogenicity [151]. Alphamab Oncology developed KN046, a recombinant PD-L1/CTLA-4 bispecific Nb-Fc fusion protein, which integrates the therapeutic benefits of dual PD-L1 and CTLA-4 blockade [42]. KN046 has been extensively studied in multiple clinical trials to evaluate its safety and efficacy across various tumors (clinicaltrials.gov). Compared to the combination of anti-PD-L1 and CTLA-4 mAbs, KN046 may have potential competitive advantages in terms of cost-effectiveness and improved safety profiles. The FDA has granted orphan drug designation to a combination of KN046 and KN026 (an anti-HER2 bispecific antibody) therapy for the treatment of HER2-positive or low-expression gastric or gastroesophageal junction cancer [152]. Similarly, Zeng et al. developed Z15-0, a bispecific nanobody construct targeting both PD-1 and CTLA-4, which incorporates an Fc domain to enhance its functionality. This construct effectively blocked the coinhibitory signaling pathways of PD-1 and CTLA-4, resulting in significant tumor growth suppression and TME remodeling in hCTLA-4/hPD-1 transgenic C57BL/6 mouse models [153]. Beyond established immune checkpoint targets, researchers have expanded their focus to develop inhibitors against recently identified immune checkpoint molecules, such as TIGIT, which plays a critical role in regulating T cell and NK cell activity. In a study, a recombined multivalent bispecific antibody was engineered through the fusion of four TIGIT-specific nanobodies, targeting two distinct epitopes, to a tetravalent PD-L1-Fc. The recombined bispecific antibody not only retained potent inhibitory capacity against both PD-1/PD-L1 and TIGIT/CD155 signaling pathways, but also demonstrated a synergistic enhancement of T cell responses in vitro, compared to its parental nanobodies [154]. In another study, Li et al. engineered a bispecific nanobody (BsNb PX4) targeting CXCR4 and PD-L1 to reprogram the pancreatic tumor microenvironment via selective disruption of the SDF-1/CXCR4 signaling axis. BsNb PX4 demonstrated dual therapeutic effects by suppressing epithelial-to-mesenchymal transition (EMT) in pancreatic cancer cells and significantly augmenting tumor-infiltrating lymphocyte (TIL) populations within the tumor microenvironment [155].

### Targeted delivery of nanobody-based ICIs

Although ICIs have demonstrated significant clinical benefits, their application is often limited by immune-related adverse events (irAEs) [131]. To address this challenge, there is a growing interest in improving the localized delivery of ICIs directly into the TME. CAR-T cells, endowed with tumor-targeting abilities and prolonged persistence in the TME, represent an optimal platform for the localized delivery of ICIs. Given the small size and stability of nanobodies, engineering CAR-T cells to secrete nanobodies targeting immune checkpoints serves as a promising strategy. In a pioneering study, Xie et al. generated nanobody-based CAR-T cells capable of secreting immune-modulating nanobodies and nanobody-Fc fusions and evaluated their therapeutic potential in syngeneic mouse models. The successful detection of secreted nanobodies confirmed the feasibility of this approach. These ICI-secreting CAR-T cells demonstrated improved antitumor efficacy, persistence, and safety [156]. By delivering immune checkpoint blockade nanobodies directly into the TME, this strategy maximizes the therapeutic advantages of both CAR-T cells and ICIs while minimizing systemic side effects. Currently, several registered Phase 1 clinical trials are underway to assess the safety and efficacy of anti-PD-1 nanobody-secreting MSLN-CAR-T cells in patients with MSLN-positive advanced solid tumors (NCT04503980, NCT04489862 and NCT05089266). Consistent with these findings, Petit et al. proposed a localized delivery strategy using tumor-targeting T cells to secrete anti-PD-L1 nanobodies at the tumor site. Compared to systemic administration of anti-PD-L1 antibodies, this approach demonstrated enhanced tumor control while minimizing systemic toxicity risks [157]. Recent studies have also highlighted the role of CD39, a key enzyme of the ATP-adenosine pathway, in shaping the immunosuppressive TME. The anti-CD39 nanobody demonstrated the ability to mitigate adenosine-driven immune exhaustion and enhance T cell cytotoxicity against AML [158]. In another study, the anti-CD39 nanobody enhanced the functionality of MSLN CAR-T cells in mouse models. Notably, the anti-CD39 nanobody-secreting MSLN CAR-T cells demonstrated superior antitumor efficacy compared to the combination of MSLN CAR-T cells and systemic infusion of CD39-specific nanobodies [159].

In addition to CAR-T cells, various delivery platforms, such as viruses, bacteria, lipid nanoparticles (LNPs), and metal-organic frameworks, have been explored for their potential to locally deliver nanobodies. Among these, adeno-associated virus (AAV), a well-characterized gene delivery vector with a proven safety profile, has been employed to provide sustained expression of anti-PD-1 antibodies within tumors [160]. CD47, a molecule expressed on tumor cells, sends a “don’t eat me” signal, helping tumors evade immune attacks. However, blocking CD47 alone is often insufficient to trigger a potent immune response. To address this limitation, Li et al. engineered an oncolytic vaccinia virus platform (OVV-αCD47nb) with integrated nanobody secretion capability specifically targeting the CD47 signaling axis. Intratumorally administration of OVV-αCD47nb reshaped the immunosuppressive TME and promoted systemic antitumor activity by boosting both innate and adaptive immune responses [161]. To achieve precise delivery of immune checkpoint blockade nanobodies, Gurbatri et al. designed a probiotic-based bacteria system. A genetically modified strain of *Escherichia coli*, termed SLIC, was designed to express PD-L1- and CTLA-4-specific nanobodies. The bacteria selectively grew in the necrotic core of tumors, where they lysed to release nanobodies continuously and effectively within the TME. Compared to the combination of clinically relevant anti-PD-L1 and CTLA-4 antibodies, a single intratumoral or systemic injection of the engineered probiotic system demonstrated enhanced therapeutic effect on tumors in syngeneic mouse models. Importantly, the bacteria were cleared upon tumor regression, enhancing the safety profile of this approach [162]. These findings highlight the potential of probiotic-based systems as safe and effective platforms for combined immunotherapy. Currently, LNPs represent the widely used formulation for mRNA delivery, facilitating targeted antibody or protein production. The LNP-mRNA platform has been employed to deliver optimized mRNA sequences encoding Z15-0. The Z15-0-2 LNP-mRNAs demonstrated dose-dependent efficacy in suppressing tumor growth in murine models, with a marked elevation in T and NK cell populations within the tumor microenvironment and corresponding changes in cytokine profiles observed post-treatment [153]. In another innovative approach, Jiang et al. used ZIF-8, a widely studied nano-delivery system, to encapsulate KN046, forming KN046@^19^F-ZIF-8. Under normal physiological conditions, ZIF-8 remains stable, protecting KN046 from premature release. However, in the weakly acidic and glutathione-rich tumor microenvironment, ZIF-8 rapidly degrades, releasing KN046 to simultaneously block PD-L1 and CTLA-4 signaling pathways. Thus, KN046@^19^F-ZIF-8 significantly enhanced antitumor activity while minimizing off-target effects, demonstrating the feasibility of ICIs locally using smart delivery agents [163]. In a novel chemo-immunotherapeutic strategy, Zhao et al. co-delivered a nanobody-catalyst conjugate and a doxorubicin (DOX) prodrug. In this approach, an anti-PD-L1 nanobody was conjugated with a ruthenium catalyst Ru-MI, forming the Ru-PD-L1 complex. Upon binding to PD-L1^+^ tumor cells, the Ru-PD-L1 facilitated the activation of the DOX prodrug, inducing immunogenic cell death. Concurrently, PD-L1 blockade by the nanobody enhanced T-cell activity by alleviating the immunosuppressive TME, synergistically enhancing tumor cell killing and anti-tumor immunity [164].

## Nanobody-based immune cell engagers

**Fig. 5:**
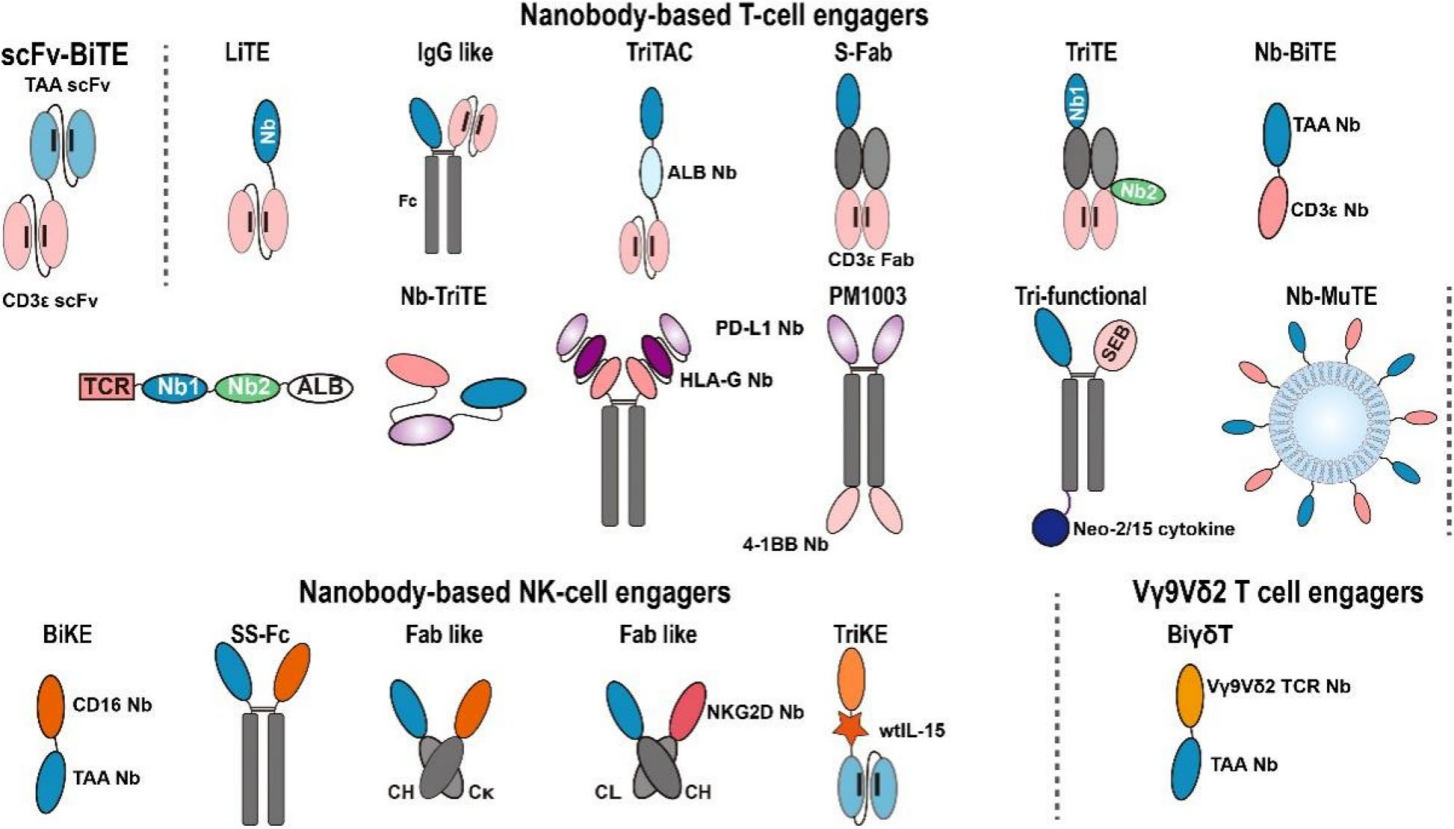
Schematic overview of diverse nanobody-based formats for immune cell engagers. The bispecific T cell engager architecture fundamentally comprises dual scFv modules, with one domain specifically engaging CD3ε on T lymphocytes while the other selectively binds to TAAs. Nanobody-based immune cell engagers, designed in various formats, redirect immune cells, including T cells, NK cells and Vγ9Vδ2 T cells, to tumor cells

Unlike mAbs that target a single antigen, immune cell-engaging antibodies are recombinant proteins designed to bind both immune cells and TAAs, enabling selective tumor cell elimination. Among these, BiTEs represent a prominent class in cancer immunotherapy [165, 166]. BiTEs are engineered as scFvs linked by a small linker, with one scFv targeting CD3 on T cells and the other targeting a TAA, facilitating the formation of lytic immune synapses and subsequent tumor cell lysis [165]. Blinatumomab, which targets both CD3 and CD19, achieved substantial clinical efficacy in hematologic malignancies and was the first FDA-approved BiTE [165]. Building on this success, the design principles of BiTEs have been extended to develop bispecific and trispecific antibodies that engage various immune cells, including T cells, natural killer (NK) cells, and γδ T cells, to enhance targeted immune activation within the TME [167–170]. Owing to their unique properties, nanobodies have also been extensively utilized in the construction of bispecific and trispecific antibodies, further expanding the potential of multifunctional immune cell-engaging therapies (Fig. 5).

### T cell-engaging bi/tri-specific antibodies

To improve the properties of traditional BiTEs, researchers modified their structure in various formats (e.g., S-Fab, IgG-like, LiTE). To further reduce the molecular size and enhance tumor penetration, they replaced the TAA-targeting scFv with nanobodies. These bispecific antibodies demonstrated the ability to induce T cell-mediated tumor cell killing [171–174]. In a recent study, Zhang et al. engineered a bispecific antibody composed of a B7H3-specific nanobody and a CD3-specific scFv, employing the oncolytic virus (OV) as a delivery system. The recombinant OV triggered enhanced immune responses against tumors and reshaped the suppressive TME in immunocompetent mouse models [175].

By replacing the two scFvs in traditional BiTEs with nanobodies, Yang et al. developed a novel nanobody-based bispecific T-cell engager platform. Based on this platform, they constructed CD105-CD3/Nb-BiTE, which demonstrated enhanced T-cell activation and tumor-killing efficacy both in vitro and in vivo [176]. To treat PD-L1 positive melanoma, Li et al. designed a nanobody-based PD-L1xCD3 BiTE, capable of simultaneously disrupting the PD-L1/PD-1 interaction and activating T cells. This PD-L1xCD3 BiTE showed potent killing activity against melanoma cells in a PD-L1-dependent manner, with an IC50 of 1.212 μg/ml [177]. A novel bispecific T cell engager SAR444200, incorporating a GPC3-specific nanobody and a TCRαβ-targeting moiety, was designed to selectively redirect T cell cytotoxicity toward GPC3-expressing malignancies. In a phase 1/2 open-label trial (EudraCT 2021-006623-17/NCT05450562), SAR444200 demonstrated a favorable safety profile in patients with advanced GPC3^+^ solid tumors [178]. Given the role of 4-1BB as a potent activator of both T cells and NK cells, its combination with ICIs has been explored to further enhance antitumor immune responses [179, 180]. Zhai et al. reported PM1003, a nanobody-based PD-L1/4-1BB bispecific antibody. PM1003 not only blocks the PD-1/PD-L1 interaction and but also activates T cells through 4-1BB-mediated signaling. The anti-PD-L1 nanobody in PM1003 targets binding epitopes similar to the approved anti-PD-L1 mAbs, such as atezolizumab, avelumab and durvalumab. In contrast, the anti-4-1BB nanobody binds to a unique epitope within the membrane-proximal cysteine-rich domain 4 (CRD) of 4-1BB, distinct from other approved anti-4-1BB mAbs. This unique binding site confers strong potency and low toxicity to the anti-4-1BB nanobody. Importantly, both these binding domains retain their functional activity in the bispecific format. PM1003 inhibited tumor growth with minimal hepatotoxicity in murine models, outperforming both the combination of its individual components and Urelumab. Additionally, PM1003 was shown to induce immune memory in mice [181]. Based on these promising results, a phase 1/2a clinical trial of PM1003 in patients with advanced solid tumors has been initiated in China (ChiCTR2100052887).

To endow nanobody-based BiTEs with superior functionality, structural modifications and optimizations have been implemented in their backbone architecture. Harpoon Therapeutics has developed an innovative trispecific T cell activating construct (TriTAC) platform. This novel construct comprises three functional components: a CD3ε-specific scFv for T cell activation, a TAA-binding nanobody for target recognition, and a human serum albumin (HSA)-specific nanobody for half-life extension. With a molecular weight of approximately 53 kDa, the TriTAC demonstrates superior characteristics compared to conventional half-life extended T cell engagers using an Fc domain, including enhanced potency in inducing targeted T cell-mediated cytotoxicity, which may translate into improved therapeutic efficacy and safety profiles in solid tumor treatment [43, 182]. The modular design of the TriTAC platform enables its application across various malignancies through interchangeable antigen-binding nanobodies. Several TriTAC-based therapeutics have advanced into clinical development: BCMA-targeting HPN217 (NCT04184050), MSLN-targeting HPN536 (NCT03872206), DLL-targeting HPN328 (NCT04471727) and PSMA-targeting HPN424 (NCT03577028) [43, 182–184]. Notably, HPN217 has obtained fast track designation from the FDA to treat R/R multiple myeloma. In another study, Ding et al. engineered a novel nanobody-based trispecific T cell engager (Nb-TriTE) by incorporating an anti-PD-1 nanobody into a FAP-targeting Nb-based BiTE. This innovative construct demonstrated dual functionality: mediating lysis of FAP^+^ cancer-associated fibroblasts while enhancing T cell activation through overcoming tumor-mediated immunosuppression [185]. Given the synergistic nature of immune checkpoint pathways in tumor immune evasion, single-target approaches against PD-1/PD-L1 have shown limited therapeutic efficacy and are prone to resistance development. To address this limitation, an advanced Nb-TriTE was developed by integrating PD-L1-, HLA-G-, and CD3-specific nanobodies with a human Fc domain. This trispecific engager exhibited enhanced tumor-killing capacity in vitro and in humanized orthotopic lung cancer mouse models by redirecting immune cells to the tumor regions and strengthening their cytotoxicity [186]. To address tumor heterogeneity, a widely adopted approach is to engineer T cells with multi-antigen targeting specificity. Liu et al. developed a novel strategy by engineering a HER2/VEGFR2/CD3 trispecific antibody (tsAb) through site-specific fusion of VEGFR2- and HER2-targeted nanobodies with a CD3-specific Fab. Compared with the bispecific counterparts, the structurally optimized tsAb demonstrated prolonged half-life and enhanced antitumor efficacy against Her2- and/or VEGFR2-expressing tumors, especially in the Herceptin- and Cyramza-resistant tumor models [187]. To address the challenges of antigen escape, limited plasma half-life, and treatment-related toxicity in AML therapy, Zeng et al. designed an innovative dual-targeting T cell engager (CD33/CD123 NANOBODY TCE) using the NANOBODY platform. The construct, designed in a beads-on-a-string configuration, incorporated CD33-, CD123-, and TCRαβ-specific nanobodies conjugated with human serum albumin. The CD33/CD123 NANOBODY TCE exhibited superior therapeutic potential, demonstrating: (1) enhanced cytolytic activity against CD33^+^/CD123^+^ leukemic cells compared to single-target TCEs; (2) favorable pharmacokinetic and pharmacodynamic profiles in nonhuman primate models with minimal toxicity; and (3) effective induction of apoptosis in both bulk AML blasts and CD34^+^ leukemic stem cells [188]. Yu et al. engineered a novel multivalent immune cell engager incorporating multiple functional domains: (1) a tumor-targeting nanobody for specific antigen recognition, (2) a superantigen variant for simultaneous NK and T cell activation, (3) a Neo-2/15 cytokine moiety for enhanced immune stimulation, and (4) an Fc domain for pharmacokinetic optimization. This innovative construct demonstrated potent anti-tumor efficacy across various tumor models by effectively redirecting and activating both NK and T cells within the tumor microenvironment [189]. In another study, Xie et al. developed an innovative nanobody-derived multifunctional T cell engager platform through the conjugation of tumor-targeting and T cell-engaging nanobodies to liposomal nanoparticles, denoted as Nb-MuTE. The Nb-MuTE significantly promoted tumor-infiltrating lymphocyte recruitment and potentiated T cell-mediated antitumor responses, representing a promising option for T cell-based immunotherapy [190].

### NK-cell engaging antibodies

As essential effectors of innate immunity, natural killer cells serve fundamental functions in maintaining immunological homeostasis and mediating antitumor responses. These cells kill tumor cells through multiple mechanisms, including antibody-dependent cell-mediated cytotoxicity (ADCC) [170, 191, 192]. Key activation receptors expressed on NK cells, such as CD16a (FcγRIIIa) and NKG2D, provide essential signals to trigger NK-cell mediated cytotoxicity [170, 193]. To harness the therapeutic potential of NK cells, bispecific antibodies have been generated by linking an anti-CD16a nanobody and an anti-TAA nanobody. These nanobody-based bispecific antibodies effectively redirect NK cells to lyse tumor cells, while exhibiting high solubility, stability, and ease of production in *Escherichia coli* [194–199]. In a similar approach, Raynaud et al. constructed bispecific anti-NKG2D/HER2 nanobodies in a Fab-like format to induce activation and cytotoxicity of unprimed NK cells. Since these bispecific antibodies did not compete with NKG2D ligands, they may retain functionality even in the presence of soluble NKG2DL which is often associated with immunosuppressive effects [200].

Beyond bispecific antibodies, the trispecific killer engager (TriKE) platform has been developed to enhance antigen-dependent NK cell activation and expansion. First-generation TriKE molecules consist of two scFvs, one targeting CD16 and the other targeting a TAA, linked to an IL-15 moiety that promotes NK cell activation and survival [201]. A CD33-targeting TriKE has advanced to clinical trials (NCT03214666). However, first-generation TriKEs faced limitations due to the inactivity of wild-type IL-15 (wtIL-15) and the reduced activity of mutated IL-15 (mIL-15) compared to recombinant human IL-15 (rhIL-15). To address this challenge, the anti-CD16 scFv was replaced with a humanized anti-CD16 nanobody, significantly improving the affinity of TriKE and enabling wtIL-15 to exhibit activity comparable to rhIL-15. This structural optimization culminated in the development of second-generation TriKEs, integrating three functional components: an anti-CD16 nanobody, wtIL-15, and an scFv against TAAs. Compared to their first-generation counterparts, second-generation CD33 TriKEs demonstrated superior NK cell activation, expansion, and tumor control in vitro and in vivo [202]. Similarly, a CLEC12 A-targeting second-generation TriKE molecular exhibited potent NK-cell mediated cytotoxicity with limited on-target off-tumor toxicity [203]. The second-generation TriKE is poised to supersede the first-generation TriKE in the treatment of cancer patients, offering enhanced therapeutic potential. In an innovative approach, NK cells engineered to express 5-azido sialic acid were conjugated with an aza-dibenzocyclooctyne-modified EGFR-specific nanobody via bioorthogonal click chemistry, demonstrating potent cytotoxicity against EGFR-positive tumors [204]. The advancement of readily available, standardized NK cell therapeutics, when integrated with the adaptable nature of nanobody-mediated functional enhancement approaches, presents substantial opportunities for broadening the clinical application of tumor immunotherapy [205–207].

### γδ T cell-engaging antibodies

γδ T cells, a small subset of T cells expressing γδ TCRs, exhibit innate immunity-like functions. Within this population, Vγ9Vδ2-T cells constitute the major circulating lymphocyte subset, representing the most abundant γδ T cell population in peripheral circulation. Unlike conventional αβ T cells, Vγ9Vδ2-T cells are activated by phosphoantigens (pAgs), which are produced by cellular pathogens and accumulated in malignant cells. Their MHC-independent recognition mechanism and potent antitumor activity make Vγ9Vδ2-T cells a promising candidate for cancer immunotherapy [208, 209]. Vγ9Vδ2-T cell therapies were generally well tolerated and elicited antitumor responses in some patients, however, their clinical efficacy remains inconsistent [210]. One potential approach to improve the antitumor activity involves augmenting the functional activation and tissue penetration of Vγ9Vδ2 T cells in the TME. γδ T cell-engagers address these challenges by offering distinct advantages over conventional CD3-based T cell engagers: they selectively activate Vγ9Vδ2-T cells, reducing Treg-mediated suppression; synergize with pAgs to enhance cytotoxicity, overcoming tumor antigen loss; amplify immune responses by activating αβ T cells and dendritic cells; and enable allogeneic infusion with low GVHD risk [209, 211–215]. To implement this strategy, Van der Vliet et al. developed γδ T cell-engagers by immunizing llamas with human Vγ9Vδ2-T cells, thereby isolating anti-Vγ9Vδ2-TCR nanobodies with high specificity and potent activation function. By combining the anti-Vγ9Vδ2-TCR nanobody with a nanobody against TAAs, several bispecific antibodies were developed [209, 211–213]. For instance, a bispecific antibody incorporating an antagonistic anti-EGFR nanobody and the agonistic anti-TCR nanobody efficiently promoted Vγ9Vδ2-T cells to produce pro-inflammatory cytokines and kill target cells, independent of KRAS or BRAF mutations in EGFR signal pathway. The application of Vγ9Vδ2-T cell-engaging bispecific antibodies has also been explored in hematological malignancies. Bispecific antibodies targeting CD40 or CD1 d demonstrated the ability to activate patient-derived Vγ9Vδ2-T cells, inducing lysis of autologous tumor cells at low effector-to-target ratios. Notably, the efficacy of CD1 d-targeting bispecific engagers could be further enhanced by aminobisphosphonates. The combination of bispecific Vγ9Vδ2-T cell engagers and pAgs synergistically amplified Vγ9Vδ2-T cell-mediated cytotoxicity, suggesting a promising therapeutic strategy applicable to a wide range of tumor types.

## Other nanobody-based immune modulators

Nanobodies have been engineered into diverse immune-modulating formats beyond direct immune cell engagement or checkpoint blockade. These innovative strategies leverage nanobodies' unique properties to indirectly enhance antitumor immunity through multiple mechanisms.

### Nanobody-based immune modulators for antigen delivery and effector enhancement

Bouma et al. designed a novel cancer vaccination approach by conjugating antigen peptide-loaded liposomes with CD169- or DC-SIGN-specific nanobodies, enhancing DC-mediated antigen presentation and specific CD8^+^ T cell priming [216].

To restore Fc-triggered immune functions, Liu et al. fused a CD70-specific nanobody with the IgG-binding domain of a streptococcal protein G-derived protein, resulting in a recombinant protein (Nb3B6-C3 Fab) with a 39-fold increased serum half-life and potent Fc-mediated tumor killing [217]. Similarly, a B7-H3-specific nanobody targeting a unique epitope, when converted to an Fc-enhanced IgG, exhibited strong ADCC against colorectal cancer cells [218]. Hong et al. further enhanced Fc-mediated anticancer immune functions and half-life by conjugating a nanobody-based bispecific anti-EGFR-HER2 antibody with rhamnose (Rha) hapten, enabling endogenous anti-Rha antibodies recruitment and immune activation. The bispecific antibody preserved co-targeting ability, prolonged half-life, and triggered Fc-mediated immune functions, leading to tumor growth inhibition in tumor mouse models [219]. Additionally, multivalent Rha conjugation to an anti-EGFR nanobody activated robust Fc effector immunity, demonstrating superior antitumor efficacy and overcoming cetuximab resistance [220]. For tumor-specific IL-2 delivery, an anti-VEGFR2 nanobody was fused with a mutant IL-2, creating a novel immunocytokine that retained antigen-selective targeting, activated peripheral blood mononuclear cells (PBMCs), and extended IL-2 half-life [221].

### Nanobody-based targeted radionuclide therapies

Targeted radionuclide therapy (TRNT) delivers α-, β-, or Auger-emitting radionuclides to tumors via specific carriers, inducing localized cytotoxicity while sparing normal tissues [222, 223]. Beyond direct DNA damage, TRNT can also modulate the TME and enhance antitumor immunity [224–227]. Although two antibody-based TRNT agents have been FDA-approved, their clinical use is limited by suboptimal tumor penetration and off-target radiation exposure [228]. Nanobodies, characterized by small size, rapid clearance, and high specificity, represent an ideal alternative for next-generation TRNT strategies [17, 18].

The extensively studied HER2-targeted nanobody 2Rs15 d, when labeled with ^177^Lu, ^131^I, ^225^Ac, or ^213^Bi, has demonstrated potent inhibition of HER2-positive tumors, including trastuzumab-resistant and metastatic models [229–232]. Its efficacy was enhanced through combination with trastuzumab or Gelofusin to reduce renal retention. Compared to trastuzumab-based TRNT, radiolabeled 2Rs15 d showed faster systemic clearance and lower off-target toxicity [229]. A phase I clinical trial with ^131^I-labeled 2Rs15 d confirmed its safety and selective tumor uptake in patients with metastatic breast cancer [233].

In addition to HER2, nanobody-based TRNT platforms targeting other tumor antigens (such as CD20, CS1, fibroblast activation protein-α and macrophage targets) have exhibited both direct radiation-induced cytotoxicity and immune activation, including increased CD8⁺ T cell infiltration and PD-L1 upregulation [226, 234, 235]. These immunomodulatory effects support the rationale for combining TRNT with ICI to improve therapeutic efficacy.

Beyond radionuclide delivery, nanobodies can also serve as versatile carriers for a broad range of cytotoxic payloads, including toxins such as Pseudomonas exotoxin, chemotherapeutic drugs such as doxorubicin, photothermal agents, photosensitizers, and proteolysis-targeting chimeras (PROTACs), thereby expanding their therapeutic potential beyond direct immune modulation [236–240].

## Conclusions and perspectives

As a new pillar of cancer treatment, immunotherapy has emerged as a paradigm for the treatment of various malignancies. Among the diverse immunotherapeutic strategies, antibody-based therapies, such as CAR-T cells and ICIs, have played a pivotal role in advancing cancer treatment. Nanobodies, with their unique physiochemical properties, have significantly expanded the toolkit for drug development and aroused great interest in the field of immunotherapy. Advances in technology and economic development have accelerated the translation of nanobody-based immuno-oncology agents from bench to bedside, with several candidates, such as cilta-cel and KN035, already approved for clinical use or in advanced stages of clinical trials.

Despite these remarkable achievements, challenges persist in cancer immunotherapy. Key issues include the identification of optimal therapeutic targets and the efficient trafficking of effector cells into the suppressive TME. The discovery of novel tumor-specific antigens is critical for developing antibody drugs and minimizing systemic toxicity and side effects. As nanobody-based cell therapies continue to advance through preclinical studies and clinical trials, efforts to optimize drug structures targeting specific antigens are actively underway. Furthermore, molecular genetic and chemical modulations of nanobodies and their derivates hold promise for refining administration methods, improving antitumor activity, and reducing immune-related side effects. The inherent advantages of nanobodies, such as small size, superior tissue permeability and modular structure, enable them seamless integration with other functional elements to design multifunctional therapeutics. Beyond their role in antibody-based cell therapies, nanobodies can be conjugated with radionuclides, drugs, PROTACs, photothermal agents, or photosensitizers to enhance tumor-targeting specificity and therapeutic efficacy. This integration synergizes the capabilities of radionuclides for localized radiotoxicity, drugs for direct cytotoxicity, PROTACs for targeted protein degradation, photothermal therapy (PTT), and photodynamic therapy (PDT) for spatiotemporally precise treatment, promoting immunogenic cell death and amplifying systemic antitumor immune responses. These properties position nanobodies as versatile tools with vast potential in immunotherapy, particularly for solid tumors, which have historically been more challenging to treat with conventional therapies.

In summary, nanobodies represent a promising frontier in cancer immunotherapy, with broad application prospects across diverse cancer types. With ongoing advancements, nanobody-based immuno-oncology therapies are set to become increasingly pivotal in redefining cancer treatment landscapes, providing renewed optimism for cancer patients.

## Data Availability

No datasets were generated or analysed during the current study.

## Abbreviations

ADCC: Antibody-dependent cell-mediated cytotoxicity
ALL: Acute lymphoblastic leukemia
AML: Acute myeloid leukemia
APC: Antigen-presenting cell
BCMA: B cell maturation protein
BiTE: Bispecific T engager
CAR-T: Chimeric antigen receptor T cell
CDR: Complementarity determining region
CRD: Cysteine-rich domain
CRS: Cytokine release syndrome
CTL: Cytotoxic T cell
CTLA-4: Cytotoxic T lymphocyte antigen-4
DC: Dendritic cell
dMMR/MSI-H: Deficient mismatch repair or microsatellite instability-high
FDA: Food and drug administration
FGFR: Fibroblast growth factor receptor
FR: Framework region
Gly: Glycine
GPC: Glypican
GVHD: Graft-versus-host disease
HcAb: Heavy chain antibody
HER2: Human epidermal growth factor receptor
HSA: Human serum albumin
ICI: Immune checkpoint inhibitor
mAb: Monoclonal antibody
Leu: Leucine
MHC: Major histocompatibility complex
MUC1: Mucin 1
Nb: Nanobody
NCT: National clinical trial
NK cell: Nature killer cell
NMPA: National medical products administration
ORR: Overall response rate
pAg: Phosphoantigens
PD-1: Programmed cell death protein-1
PDX: Patient-derived xenograft
PNE: Peptide neo-epitope
PSMA: Prostate-specific membrane antigen
R/R: Relapsed or refractory
scFv: Single-chain variable fragment
sCR: Stringent complete response
sdAb: Single-domain antibody
TAA: Tumor-associated antigen
TAG: Tumor-associated glycoprotein
TCR: T cell receptor
TIL: Tumor-infiltrating lymphocyte
TM: Target module
TNP: 2,4,6-Trinitrophenyl
TriKE: Tri-specific killer engager
TriTAC: Trispecific T cell activating construct
Trp: Tryptophan
Val: Valine
VH: Light chain variable domain
VHH: Variable domain of heavy chain of heavy-chain antibody
VL: Heavy chain variable domain
